# Supplementary figures and images for: Citrullination profile analysis reveals peptidylarginine deaminase 3 as an HSV-1 target to dampen the activity of candidate antiviral restriction factors
Source: PLoS Pathog. 2023 Dec 6;19(12):e1011849. doi: 10.1371/journal.ppat.1011849 (PMC10727434; doi:10.1371/journal.ppat.1011849)

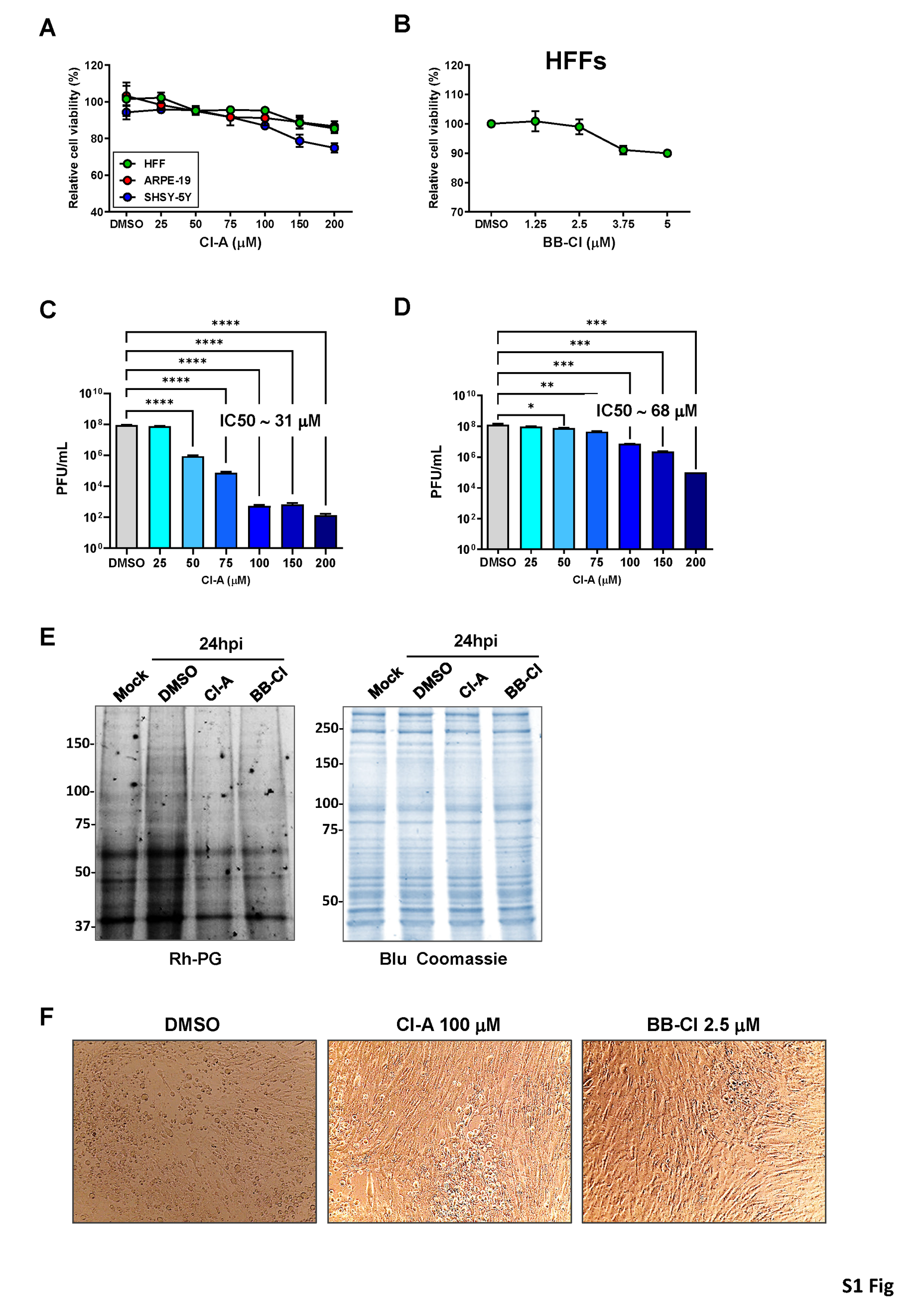

Supplement: S1 Fig — Uninfected HFFs, ARPE-19, and SHSY-5Y were treated with the indicated concentrations of Cl-A (A) and BB-Cl (B) for 24 h, and the number of viable cells was determined for each concentration using the MTT assay. Values are expressed as means ± SEM of three independent experiments. SH-SY5Y (C) and ARPE-19 (D) were infected with HSV-1 (MOI 1 PFU/cell) and then treated with increasing concentrations of Cl-A, which were given 1 h prior to virus adsorption and kept throughout the whole experiment. At 24 hpi, viral plaques were microscopically counted, and the number of plaques was plotted as a function of inhibitor concentration. Values are expressed as means ± SEM (error bars) of three independent experiments, *P < 0.05, **P < 0.01, ***P < 0.001; one-way ANOVA followed by Bonferroni’s post-test. (E) Protein lysates from uninfected (mock) or infected HFFs (24 hpi) at an MOI of 1 PFU/cell treated with Cl-A (100 μM), BB-Cl (2.5 μM) or vehicle (DMSO) were exposed to an Rh-PG citrulline-specific probe (left panel) and subjected to gel electrophoresis to detect citrullinated proteins. Equal loading was assessed by Coomassie blue staining (right panel). (F) Representative images of infected HFFs (24 hpi) at an MOI of 1 PFU/cell and treated with Cl-A (100 μM), BB-Cl (2.5 μM), or vehicle (DMSO). (TIF) [file ppat.1011849.s001.tif]

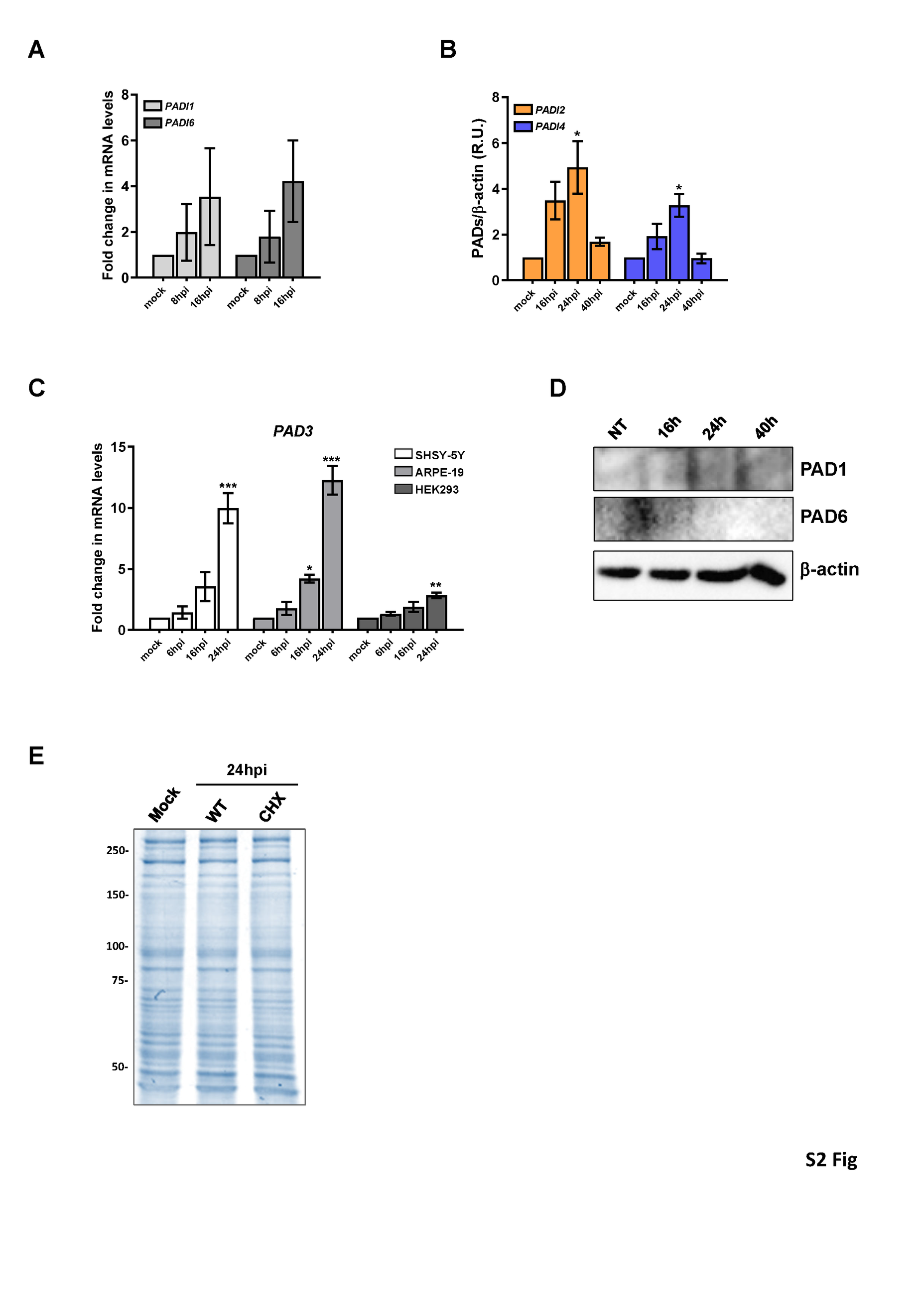

Supplement: S2 Fig — (A) mRNA expression levels of PADI isoforms by RT-qPCR of HSV-1-infected (8 and 16 hpi) vs uninfected (mock) HFFs were normalized to the housekeeping gene glyceraldehyde-3-phosphate dehydrogenase (GAPDH) and expressed as mean fold change ± SEM over mock-infected cells. *P < 0.05, **P < 0.01, ***P < 0.001; one-way ANOVA followed by Bonferroni’s post test. (B) Densitometric analysis of three independent experiments, values are expressed as fold change in PAD2 and PAD4 expression normalized to α-tubulin. (C) mRNA expression levels of PADI3 isoforms by RT-qPCR of HSV-1-infected (6, 16 and 24 hpi) vs uninfected (mock) cells were normalized to GAPDH and expressed as mean fold change ± SEM over mock-infected cells. *P < 0.05, **P < 0.01, ***P < 0.001; one-way ANOVA followed by Bonferroni’s post test. (D) Western blot analysis of protein lysates from untreated (NT) or IFN-β treated (500 U/mL) HFFs using antibodies against PAD1 or PAD6. β-actin cellular expression was used for protein loading control. One representative gel of three independent experiments is shown. (E) Blu Coomassie staining of the same protein extracts used in the experiments shown in Fig 2D (protein lysates from uninfected (mock) or HSV1-infected HFFs, treated with 150 μg/ml CHX or left untreated). (TIF) [file ppat.1011849.s002.tif]

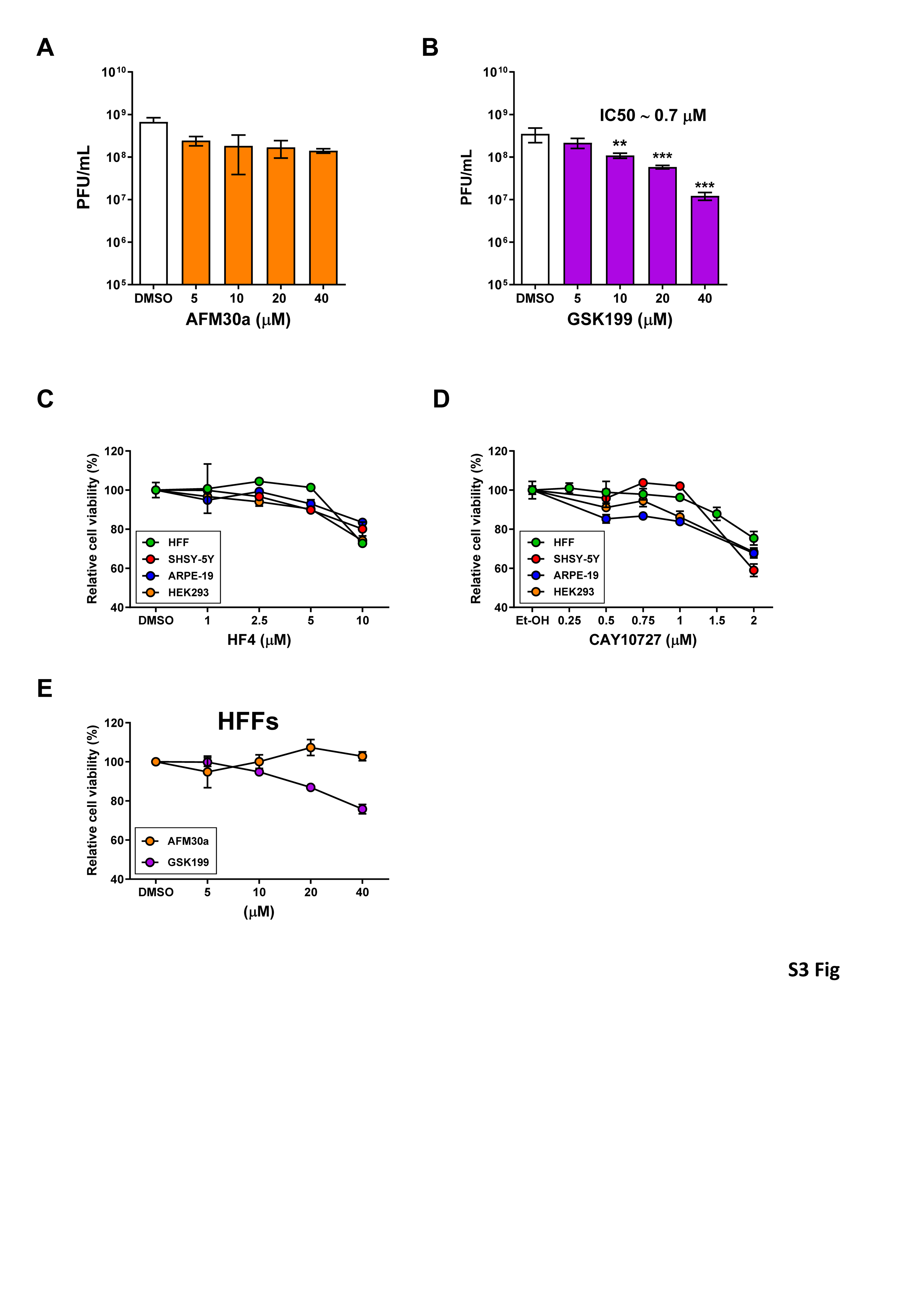

Supplement: S3 Fig — (A-B) HFFs were infected with HSV-1 (MOI 1 PFU/cell) and then treated with increasing concentrations of AFM30a (A), GSK199 (B) and CAY10727 (C), which were given 1 h prior to virus adsorption and kept throughout the whole experiment. At 24 hpi, viral plaques were microscopically counted, and the number of plaques was plotted as a function of inhibitor concentration. Values are expressed as means ± SEM (error bars) of three independent experiments, *P < 0.05, **P < 0.01, ***P < 0.001; one-way ANOVA followed by Bonferroni’s post test. (C-E) Uninfected HFF, SHSY-5Y, ARPE-19 or HEK293 cells were treated with the indicated concentrations of HF4 (C), CAY10727 (D), AFM30a, or GSK199 (E) for 24 h and the number of viable cells was determined for each concentration by MTT assay. Values are expressed as means ± SEM of three independent experiments. (TIF) [file ppat.1011849.s003.tif]

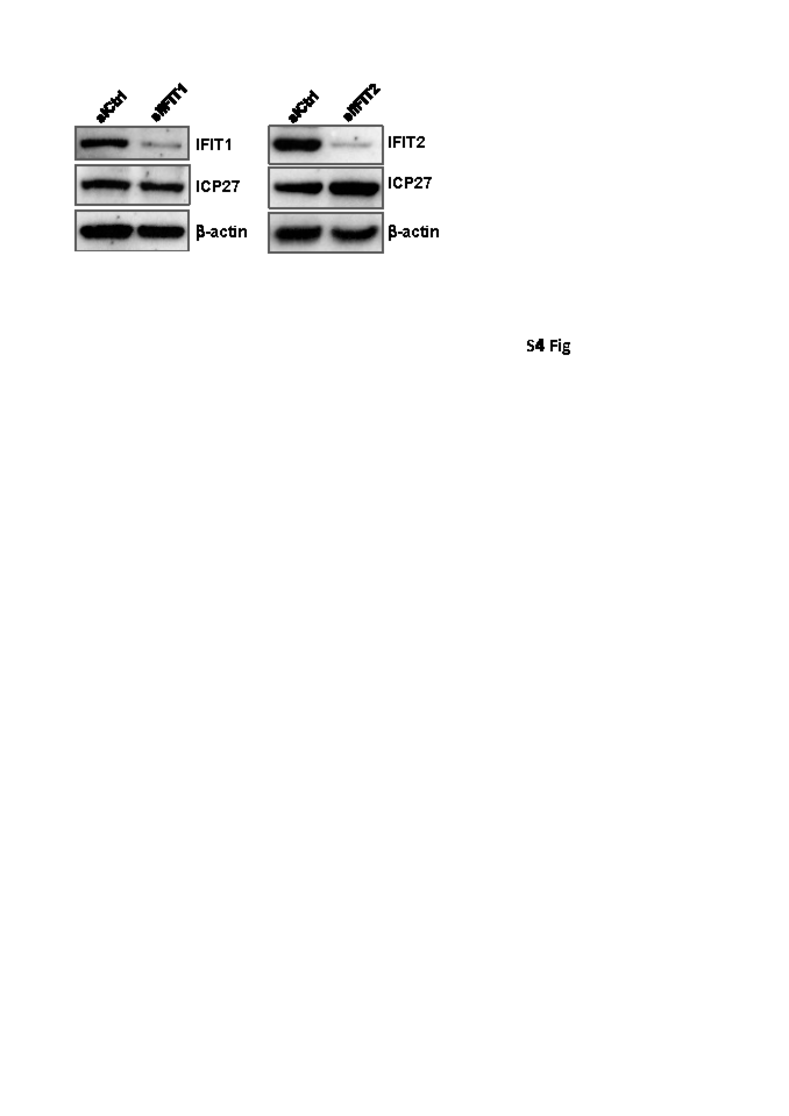

Supplement: S4 Fig — (A) The efficiency of IFIT1 or IFIT2 protein depletion at 24 hpi was assessed by immunoblotting using antibodies against IFIT1 or IFIT2, or against β-actin to check for equal loading. An anti-ICP27 antibody was also used to verify HSV-1 infection. Representative blots of three independent experiments are shown. (TIF) [file ppat.1011849.s004.tif]
