# Supplementary material for: Citrullination profile analysis reveals peptidylarginine deaminase 3 as an HSV-1 target to dampen the activity of candidate antiviral restriction factors
Source: PLoS Pathog. 2023 Dec 6;19(12):e1011849. doi: 10.1371/journal.ppat.1011849 (PMC10727434; doi:10.1371/journal.ppat.1011849)
Supplement: S1 Table — (PDF) [file ppat.1011849.s005.pdf]

Supplementary Table 1. Oligonucleotide primer sequences for qPCR and *PADI3* promoter cloning

Gene of Interest	SEQUENCE (5'-3')
GAPDH FW	AGTGGGTGTCGCTGTTGAAGT
GAPDH RV	AACGTGTCAGTGGTGGACCTG
PADI1 FW	TCCAGAGACCCTGAAGCTGT
PADI1 RV	GTGCAGCTGTCCCTGAAGAT
PADI2 FW	ACCTCCTCAGCCTCCCC
PADI2 RV	CCTACCTCTGGACCGATGTC
PADI3 FW	GCGTCCCATAGACCTCAAAC
PADI3 RV	CAGAGAATCGTGCGTGTGTC
PADI4 FW	CCTGTGGATTTCTTCTTGGC
PADI4 RV	GGGCACCTTGACTCAGCTT
PADI6 FW	CAAGGTATAGGCGTGCTGGT
PADI6 RV	TCCTCCATACCTCCAAGGAA
gE2 FW	TGTCTGTATCACCCGCAGC
gE2 RV	TTCTGGAACACCCCGCGTA
PADI3prom FW	TAACTCGAGAATGCTTTGGGGGTGCTTAT
PADI3prom RV	GGG AGCTTGCTGGTGTTGGACTTAGCTG
