# Supplementary material for: Citrullination profile analysis reveals peptidylarginine deaminase 3 as an HSV-1 target to dampen the activity of candidate antiviral restriction factors
Source: PLoS Pathog. 2023 Dec 6;19(12):e1011849. doi: 10.1371/journal.ppat.1011849 (PMC10727434; doi:10.1371/journal.ppat.1011849)
Supplement: Attachment — Submitted filename: Pasquero et al_Rebuttal.pdf [file ppat.1011849.s008.pdf]

Point-by-point reply

Please, find below the point-by-point reply to the Reviewer's comments concerning the manuscript by Pasquero *et al.* entitled "**Citrullination profile analysis reveals peptidylarginine deaminase 3 as an HSV-1 target to dampen the activity of candidate antiviral restriction factors**" (PPATHOGENS-D-23-01153).

We would like to thank the Reviewers and the Editor for their helpful comments and suggestions that helped us improve the quality of our manuscript. The changes to the last version of the manuscript have been highlighted in the text.

Major Issues: Key Experiments Required for Acceptance

Reviewer #1:

1. *It is very interesting that PAD3 protein levels are undetectable in HFF cells until after HSV-1 infection, and that HSV-1 specifically seems to need PAD3 in this cell type. However, as the authors did not test the specific PAD3 inhibitors in the other cell types included in this study (ie. the epithelial ARPE-19 cells or the SH-SY5Y neuroblastoma cells), or any other cell types for that matter, the conclusion can only be made about HFF cells. It is troublesome that there is no detectable basal levels of PAD3 in HFF cells, which begs the questions what does HSV-1 infection look like in cells with basal levels of PAD3? Clearly HFF cells express basal levels of PAD2 and 4, and HSV-1 infection appears to up regulate these isoforms as well, but with different kinetics and to different levels. Would this happen with PAD3 too, in a cell type that expresses basal levels of PAD3? Is what is observed with PAD3 in HFF cells just a cell-type specific phenomenon? The authors do show that general, pan PAD inhibition inhibits HSV-1 replication in additional cell types, but the conclusions about the PAD3 isoform specifically would be made stronger if the qPCR and westernblot analysis was done in these cell types as well. It would be greatly beneficial if the authors also included a cell type that had basal levels PAD3 expression, like HEK293T cells see citation: (Pong U, et.al. 2014, https://doi.org/10.1016/j.bbamcr.2014.02.018). The same is true for the PAD3 inhibition experiments done with HF4 (lines 184-210), these experiments should be repeated in the ARPE-19 and SH-SYY5 cell types, and perhaps HEK293T cells. These experiments would address if the isoform specificity found with HSV-1 infection is simply a matter of the HFF cells themselves, or something that occurs in all HSV-1/PAD3 relevant cell types.*

We thank Reviewer #1 for pointing out these issues related to the generalizability of our findings. As requested, we have performed qPCR and Western blot analyses using the other cell lines included in our study, namely ARPE-19 and SHSY-5Y, and, as suggested by this Reviewer, HEK293T. These new data, presented in panel D of new Fig. 2, and discussed on page 8 (lines 174-179), demonstrate that PAD3 is upregulated during HSV-1 infection (MOI 1) in all the cell lines tested. Despite minor variations between the cell lines, this suggests that the phenomenon observed in HFFs may indeed extend to other cell types. Notably, the kinetics of PAD3 consistently paralleled those of the viral protein ICP27, indicating that the timing of PAD3 production is primarily influenced by the

permissiveness of the cells and, consequently, by the kinetics of viral replication rather than being driven by differences in PAD3 basal expression.

In response to the Reviewer's additional suggestion, we have assessed the impact of the PAD3 inhibitors HF4 (0.5-5 µM; see new Fig. 4E) and CAY10727(0.5-1 µM; see new Fig. 4F) on viral plaque formation in HSV-1-infected (MOI 1) SH-SY5Y, ARPE-19, and HEK293T cells (see page 11, lines 230-233). These compounds were administered 1 h before virus adsorption and kept throughout the whole experiment. After 24 h of continuous exposure to the inhibitors, we observed a dose-dependent decrease in the number of viral particles in all treated cells. Finally, we conducted a cell viability assessment using the MTT assay in all cell lines treated under the same conditions and found that the drugs were not cytotoxic at these concentrations (refer to Suppl. Fig 3C and 3D). Collectively, these results indicate that HSV-1 infection can modulate specific PAD isoforms (*e.g.*, PAD3) across various cell types.

2. *The qRTPCR data presented in Figure 2A and discussed in the text lines 152-156 is normalized to GAPDH, which is an insufficient housekeeping gene to use with HSV-1 infection. It is very well documented that GAPDH is not a sufficient housekeeping gene to use for HSV-1 infection, as this transcript is virtually degraded by the vhs protein by 6hpi. see references (Hsu et.al. 2005, https://doi.org/10.1128/JVI.79.7.4090-4098.2005, and Watson, et.al. 2007 https://doi.org/10.1186/1743-422X-4-130) Due to this reason, the data presented at 16hpi, 24hpi and 32hpi in Figure 2A is likely an artificially inflated mean fold change. Therefore, the experiment presented in figure 2A should be repeated using a different method, such as qRTPCR using a standard curve made from plasmids encoding each PAD isoform, or an RNA-seq experiment where each gene of interest can be addressed individually over time. This is particularly relevant because the data presented in Figure 2A makes it appear that the basal levels of the PAD isoforms are equal in HFF cells, and that the lack of PAD3 protein expression in this cell type is due to regulation after the transcriptional level. This is confusing for the reader, particularly when the authors go on to mechanistically address this phenomenon as stated on line 166, using cycloheximide and IFN gamma experiments, as stated on line 166. Together the cycloheximide and IFN-gamma data only demonstrate that viral protein production is required for PAD3 up regulation, and that this up regulation is independent of the IFN response. This combined with the artificially inflated qRTPCR data make it difficult to understand what is actually going on with PAD3 expression during HSV-1 infection. In order to gain mechanistic insights into PAD3 up-regulation many more experiments need to be included than what the authors have currently done. These experiments would analyze PAD3 expression at the transcriptional level, the protein translational level, the mRNA decay level, and the protein stability level. PAA experiments should also be included to determine if PAD3 up regulation is due to a viral immediate early/ early protein, or a viral late protein.*

We thank the Reviewer for keenly pointing this out. As requested, we have repeated the RT-PCRs using two other housekeeping genes, namely *pyruvate dehydrogenase kinase 1* (*PDK1*) and *beta2-microglobulin* (*B2M*), which are among the most conserved during HSV-1 infection and are widely used in this type of analysis. As shown in the figures below, regardless of the reference gene employed, we observed a consistent pattern of PAD induction, with a marked upregulation of *PADI3* gene starting from 16 hpi, even though the absolute values may show minor variations from one reference gene to another. In light of these findings, we would rather maintain Fig. 2A in its original

form given that the main message of this experiment remains unchanged and is robustly confirmed by the Western blot analyses presented in new Figures 2B and 2D.

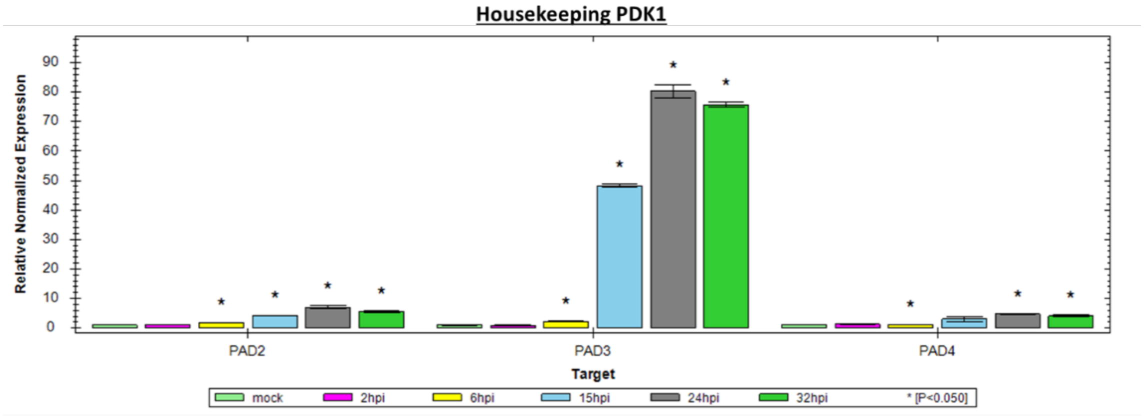

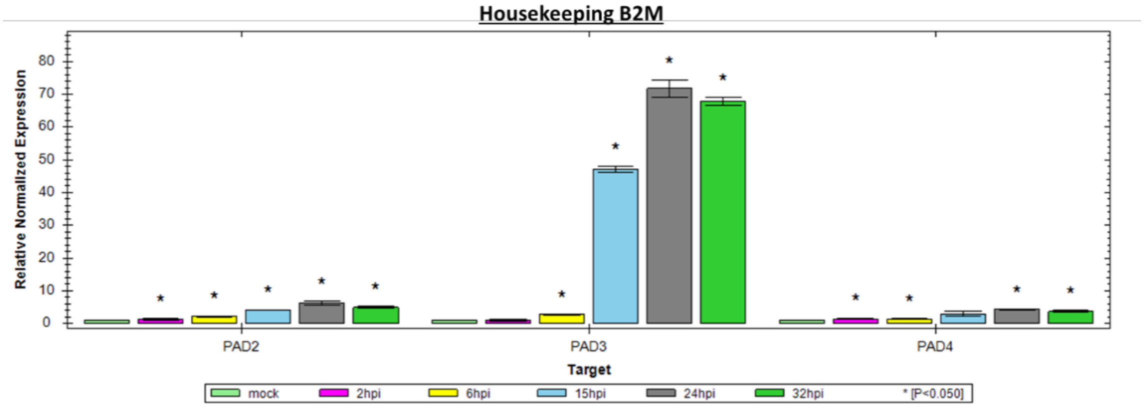

Overall, we concur with this Reviewer's observation that further data are needed to gain more mechanistic insights into PAD3 upregulation. To address this issue, we have performed initial experiment aimed to assess the promoter activity of the *PADI3* gene by dual luciferase assay. As detailed on page 9 (lines 185-190), HFFs were transiently transfected with the luciferase reporter plasmids carrying the wild-type promoter region of PAD3. Twenty-four h after electroporation, cells were infected with HSV-1. The updated Figure 3A shows that HSV-1 infection leads to a 6-fold induction of *PADI3* promoter activity, indicating that *PADI3* is upregulated at the transcriptional level through promoter activation following HSV-1 infection. Furthermore, as per the Reviewer's suggestion, we conducted experiments with the viral DNA synthesis inhibitor phosphonoformic acid (PFA, foscarnet) to determine whether PAD3 upregulation was linked to a viral immediate early/early or late protein. As expected, PFA treatment of HSV-1-infected HFFs led to a significant reduction in the synthesis of the late viral protein gD, but it had only a marginal effect on PAD3 upregulation (see new Fig. 3D and comments on page 9, lines 196-200). These combined findings, which imply that viral late proteins likely do not play a role in PAD3 transcriptional activation, have been integrated into a new Results section entitled "**PAD3 protein levels are induced through a calcium-independent HSV-1-early mechanism**". This section has been consolidated with the new results related to calcium influx (see next point and refer to pages 9-10, lines 201-210).

3. *It is well documented that HSV-1 infection causes an influx in intracellular calcium. Seeing that PAD3 expression is induced by an influx of intracellular calcium, it is possible that PAD3 up-*

regulation is a result of this rather than a direct up regulation of PAD3 gene expression by the HSV-1 viral proteins themselves. HSV-1 infection progresses though the temporal cascade much quicker than HCMV infection in HFF cells, it is possible that the influx of calcium occurs to a much greater level and to a quicker extent than in HCMV infection. This could be an alternate explanation to the results surrounding PAD3 specificity in HSV-1 infection which needs to be explored further. Experiments using artificially induced intracellular calcium levels should be included to rule out this possibility as a mechanism of action—similar to what the authors did for IFN gamma.

We are grateful to Reviewer #1 for providing us with the opportunity to address this crucial issue. Following the Reviewer's valuable suggestion, we performed experiments in which HFFs were exposed to thapsigargin (TG). TG is well-established ER stressor known for its ability to elevate cytoplasmic calcium levels by interfering with the sarcoplasmic/endoplasmic reticulum $Ca2^{+}$-ATPase (Quynh Doan *et al*., Curr Pharm Des. 2015, doi: 10.2174/1381612821666151002112824). In these experiments, we treated mock-infected HFFs with two different concentrations of TG and assessed PAD3 expression at both mRNA and protein levels after 16 h. As shown in new Fig. 3E and F, unlike HSV-1 infection (MOI 1; 16hpi), TG exposure failed to induce a significant upregulation of PAD3 at any of the concentrations applied. Of note, TG treatment of HFFs led to a substantial increase in ATF-6 expression levels (Fig. 3E), confirming its effectiveness as an ER stressor at both concentrations used. These results have been included in the new Results section entitled "PAD3 protein levels are induced through a calcium-independent HSV-1-early mechanism" (pages 9-10, lines 201-210).

Collectively, our results suggest that the overexpression of PAD3 observed during HSV-1 infection is the result of a direct transcriptional upregulation of PAD3 gene expression by HSV-1 viral proteins rather than the result of intracellular calcium influx caused by HSV-1 infection.

Reviewer #2: *None noted.*

We sincerely thank the Reviewer for his/her kind appreciation of our work and thoughtful comments.

Reviewer #3:

Figure 1F: *The 'pretreatment' arm is not really pretreatment as it overlaps with HSV addition for 2 hours. Normally pretreatment means washing off prior to viral addition and if done in that way may provide an even clearer discrepancy in panel G with 'post -treatment and a better indication of potential mechanism.*

We completely agree with the input from Reviewer #2. As suggested, we have now carried out experiments involving three different treatment schedules with Cl-A and BB-Cl (new Figs. 1F and 1G). These schedules include the one recommended by the Reviewer, where the drugs are added before infection and removed just prior to viral infection (pre-treatment, Pre). In addition, we retained the previous treatment schedules: treatments maintained for the first 2 h of infection (now referred to as Pre+), and treatments extended until 24 hpi (referred to as Post). Our results show only slight differences between the Pre and Pre+ conditions, and both failed to yield a significant reduction in

the number of viral particles when compared to the Post treatment schedule. These new results have been included on page 7 (lines 144-149). We thank the Reviewer for his/her valuable suggestion, which allowed us to deepen our understanding of the mechanism of action of PAD inhibitors.

Supplementary Fig 1F: the protein citrullination pattern according to molecular weight differs from Figure 1A. Why?

We thank this Reviewer for his/her thorough examination of our results. Regrettably, our RhPG experiments show some partial inconsistencies, primarily due to slight variations in blot running time, gel homemade preparation, and the lots of probes used. Nevertheless, we firmly believe that the essential aspects remain consistent regardless of these conditions. These include the high intensity bands observed between 37 and 50 kDa in all the samples, as well as the observed increase in signal intensity between 75 and 150 kDa during infection. These consistent data, combined with mass spectrometry data (new Fig. 5A), attests to the robustness of our results.

Figure 2c: In Discussion the authors speculate that HSV immediate early proteins are responsible for stimulating the PADs and citrullination, a key question. To differentiate IE/E proteins from late structural proteins the experiment should be repeated with foscarnet (and if confirmed, eventually with multiple IE/E protein mutants).

In line with the valuable suggestions put forth by Reviewers #1 and 3, we have duly conducted the experiments with foscarnet. Please see our response to Reviewer #1's point 3.

Part III – Minor Issues: Editorial and Data Presentation Modifications

Reviewer #1

1. *Image J should be used to quantify the data presented in the westerblots, particularly surrounding the PAD2 and PAD4 isoforms that do have basal levels in mock cells. This way all western blots, even the ones not shown in the paper could be used the quantification.*

As suggested by Reviewer #1, we have now presented the densitometric analysis to quantify the data presented in Fig. 2B relative to PAD2 and PAD4 isoforms (new Supplementary Fig. 2B)

2. *The westerblots for PAD3 in Figure 2 look underexposed in comparison to the westerblots for PAD2 and PAD4. This is concerning as the authors go to great lengths to point out that there is no PAD3 protein expression in mock infection. The difference in background color on the blots between PAD2, PAD3, and PAD4, particularly in Figure 2E, is questionable.*

We apologize for any misunderstanding that may have arisen regarding the quality of our Western blots. In all our acquisitions, we used the standard settings for automatically optimizing exposure time, both under high sensitivity and high resolution conditions. Regarding PAD3, when we manually

changed the exposure time, no bands appeared in the mock even though the existing bands became saturated, with their signals extending to cover the mock lane when the exposure times were further increased (see the figures below). We trust that this clarification, coupled with the new Western blot results presented in new Fig. 2D for the other cell lines, provides sufficient evidence to assure the Reviewer that, under our experimental conditions, PAD3 protein was undetectable by Western blots in mock-infected HFFs.

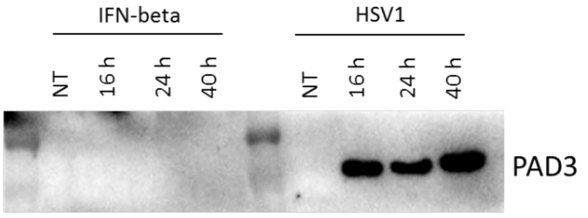

Automatically optimized exposure time

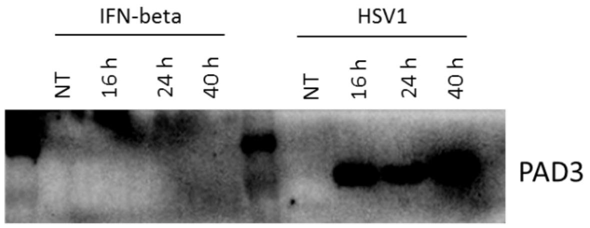

Manually overexposed

3. *The authors point out that many viral proteins were found to be citrullinated in their screen at 16 and 24 hpi, in addition to the IFIT1 and IFIT2 proteins among others in Figure 4 A and B. The authors use an IP with an anti-peptidylcitrulline antibody to pull down IFIT1 and IFIT2 to show they are citrullinated during infection. As UL54 (ICP27) was shown to be citrullinated at 16hpi in Figure 4B, the authors should be able to pull ICP27 down with the antibody used for the IP in Figure 4C. They should do this to confirm their results in their volcano plot, and it should be easy as they already have all regents required.*

We thank the Reviewer for this important comment, which significantly enhanced our citrullination profile results. In response to the suggestion, we conducted an ICP27 pull-down experiment using the same protein extract and the same antibody used for the IP of the cellular proteins IFIT1 and IFIT2. As shown in the new Fig. 5D, the IP confirmed the findings from the volcano plot, showing that viral ICP27 is somewhat undergoing citrullination at least at 16 hpi. This result has now been included in the last paragraph of the Results section (page 12, lines 270-276).

4. *The authors should add to the discussion after line 315 to speculate why there are citrullinated viral proteins during HSV-1 infection.*

We are grateful to the Reviewer for raising this interesting topic for discussion. Speculation about citrullinated viral protein were added in the new manuscript (pages 14-15, lines 326-332).

5. *The way the figures and the supplementary figures are presented is very confusing, and it makes it difficult for the reviewer/reader to find the figures that are being discussed. Many of the figures and the supplementary figures and legends have been separated. For example, Figure 1 A, B and C are presented as a separate figure from Figure 1 D, E, F and G. They each have their own legend, and therefore should be renumbered to be their own figures. The authors have done this with supplementary figures as well. The figures and supplementary figures should be re-numbered so each*

legend describes its own figure and has its own individual number. Or the panels and legends need to be combined for each number. The text for this needs to be altered as well in order to make it more logical.

We apologize for these inaccuracies, and we thank the Reviewer for helping us improve the manuscript. In this new version, the main and supplementary figures have been renumbered so that each legend describes its own figure and has its own individual number. The text has been changed accordingly. We hope that this new manuscript organization will adequately address the pointed criticisms.

6. *It is unclear if an MOI of 1 was used for every experiment presented in the paper. The methods section needs include if an MOI of 1 was used for the qRTPCR experiments the westerblots etc.*

We apologize for these inaccuracies. An MOI of 1 was used for all the experiment unless otherwise specified. Accordingly, the new Methods section includes the MOI used for every experiment.

7. *The legend for Supplementary figure 2 is missing the correct description of (A). Currently (A) describes (B) and (B) describes (C).*

The figures and legends have been amended and properly ordered.

Reviewer #2

1. *With IC50s in the micromolar range in cell culture (61 uM for C1-A), it is difficult to characterize the PAD inhibitors as potent. The authors are requested to tone this down.*

We fully agree with this Reviewer. Accordingly, we have toned down the description of the antiviral activity of Cl-amidine in the Abstract (page 2, line 33).

2.*I don't think host-directed antiviral therapies have delivered yet on their promise. They have been a popular area of research for more than a decade. There are several examples of drugs that boost the immune response non-specifically or specifically. However, there are disappointingly few antivirals approved for use in humans that target host factors critical for a virus' replication cycle. Just a comment on the potential of HTAs.*

Given the evolutionary conservation of host factors, the development of host-directed therapies (HDT) would impose a higher genetic barrier to the emergence of resistant strains. Nevertheless, it is important to point out that targeting host factors or pathways potentially may carry the risk of undesired off-target effects, thus requiring a careful risk-benefit assessment. Currently, the modulation of the innate immune response is a prevalent strategy in HTA development. For example, there are 12 interferon (IFN)-based treatments, belonging to 8 distinct IFN types approved by the FDA for the treatment of diseases with viral or malignant origin, including hepatitis B or C, genital warts, and other infections (Badia *et al*., Front Virol 2022, doi 10.3389/fviro.2022.935933/full).

Another example of HTA approved for clinical practice is Maraviroc, a negative allosteric modulator of the C-C chemokine receptor type 5 (CCR5), which inhibits HIV-1 entry and replication (Lieberman-Blum *et al.*, Clin Ther 2008, doi 10.1016/s0149-2918(08)80048-3). Furthermore, the recent COVID-19 pandemic has spurred many successful clinical trials involving HTAs. In this context, the estrogen receptor antagonist Raloxifene was evaluated in a phase 2 multicenter trial. This randomized, placebo-controlled study provided evidence of its effectiveness in managing early-stage COVID-19 among mild to moderate patients by reducing the duration of viral shedding (Nicastri et al., EClinicalMedicine 2022, doi: 10.1016/j.eclinm.2022.101450).

As our understanding of virus-host interactions deepens, we anticipate a greater emphasis on targeting critical cellular components or pathways crucial for viral fitness. This strategy will enable intervention at virtually every stage of the viral replication cycle and prove crucial in addressing viral strains resistant to direct antiviral therapies.

3.*The legend of Fig. S2 does not match the panels shown.*

We apologize for these inaccuracies; the figures and legends have been corrected and reorganized.

Reviewer #3

Supplementary Figure 2 Legend: Legend to panel A is missing so the panel numbering is out of order. Figure 3: Same heading as figure 2: Specify by siRNA.

We thank this and the other Reviewers for pointing out these oversights. Accordingly, the figures and legends have been corrected and properly ordered.

Supplementary Fig 3C: shows an important confirmation of the role of PAD3 so it should be paired with main figure 3A. Supplementary Figures 4A and B are interesting and part of the narrative and should be main figures.

We thank this Reviewer for this suggestion, which helped us improve the overall quality of our data. As suggested, we have reorganized the figures. Specifically, according to the Reviewer #1's suggestion, S3C Fig. is now new Fig. 4B, whereas Fig. 4B and 4C are now part of the new and restructured Fig. 5 (panel 5C and panel 5E, respectively).
